# Supplementary material for: Right ventricle free wall longitudinal strain screening of lung transplant candidates
Source: PLoS One. 2024 Dec 20;19(12):e0314235. doi: 10.1371/journal.pone.0314235 (PMC11661623; doi:10.1371/journal.pone.0314235)
Supplement: S1 Checklist — (DOC) [file pone.0314235.s001.doc]

STROBE Statement

Manuscript title: Right Ventricle Free Wall Longitudinal Strain Screening of Lung Transplant Candidates

|  | Item No | Recommendation | Page # |
| --- | --- | --- | --- |
| **Title and abstract** | 1 | (*a*) Indicate the study’s design with a commonly used term in the title or the abstract | Pag. 4. “In a single-center prospective observational cohort study” |
|  |  | (*b*) Provide in the abstract an informative and balanced summary of what was done and what was found | Pag. 4. See Methods and Results in the abstract |
| Introduction | | |  |
| Background/rationale | 2 | Explain the scientific background and rationale for the investigation being reported | Pag. 8 |
| Objectives | 3 | State specific objectives, including any prespecified hypotheses | Pag. 9. “We hypothesized that patients enlisted for LUTX may have impaired RVFWLS, and that RVFWLS can have better diagnostic capabilities in detecting RV dysfunction as compareed to standard two-dimensional echocardiography and ventriculography. Accordingly, this prospective observational cohort study aimed to: 1) evaluate the RVFWLS in patients listed for LUTX; 2) investigate the relationship between RVFWLS and conventional RV echocardiographic indexes.” |
| Methods | | |  |
| Study design | 4 | Present key elements of study design early in the paper | Pag. 9. “This study is a single-center prospective observational cohort analysis of consecutive patients enlisted for LUTX at an Italian tertiary referral center from January 2021 to November 2022.” |
| Setting | 5 | Describe the setting, locations, and relevant dates, including periods of recruitment, exposure, follow-up, and data collection | Pag. 9. “at an Italian tertiary referral center from January 2021 to November 2022. Due to logistical constraints subsequent to the COVID-19 pandemic, actual recruitment of patients commenced on the 1st November 2021, and ended on the 31st November 2022.” |
| Participants | 6 | (*a*) Give the eligibility criteria, and the sources and methods of selection of participants. Describe methods of follow-up | Pag. 9-10. “All patients enlisted for LUTX during the study period were considered for inclusion. Exclusion criteria were: 1) single LUTX; 2) re-transplantation; 3) patients bridged to LUTX with veno-venous ECLS; 4) incomplete medical records.” |
|  |  | (*b*) For matched studies, give matching criteria and number of exposed and unexposed | n/a. |
| Variables | 7 | Clearly define all outcomes, exposures, predictors, potential confounders, and effect modifiers. Give diagnostic criteria, if applicable | Pag. 10-11. |
| Data sources/ measurement | 8* | For each variable of interest, give sources of data and details of methods of assessment (measurement). Describe comparability of assessment methods if there is more than one group | Pag. 10-11. |
| Bias | 9 | Describe any efforts to address potential sources of bias | Pag. 10-11. Consecutive enrolment of patients. Double assessment of echocardiography. Blinding of operators regarding clinical status of the patient. |
| Study size | 10 | Explain how the study size was arrived at | Pag. 11. “Sample size calculation was performed utilizing a Wilcoxon signed-rank test (one sample case). Considering a normal value of RVFWLS of -29% ± 4.5% (12), with an α error probability of 0.05, and a power (1-β error probability) of 0.8, a reduction in RVFWLS in patients enlisted for LUTX consisting with a RVFWLS of -27 could be detected with a sample size of 35.” |
| Quantitative variables | 11 | Explain how quantitative variables were handled in the analyses. If applicable, describe which groupings were chosen and why | Pag. 11. “Data were reported as the median [first-third quartile] and number of events (percentage of the subgroup) for continuous and categorical variables, respectively.” |
| Statistical methods | 12 | (*a*) Describe all statistical methods, including those used to control for confounding | Pag. 11 |
|  |  | (*b*) Describe any methods used to examine subgroups and interactions | Pag. 11. “Data were reported as the median [first-third quartile] and number of events (percentage of the subgroup) for continuous and categorical variables, respectively.” |
|  |  | (*c*) Explain how missing data were addressed | Pag. 11 “Missing data were not imputed, and, whereas strain measurement where missing (i.e., patients with poor acoustic windows for RV evaluation), were not considered for the echocardiographic analysis.” |
|  |  | (*d*) If applicable, explain how loss to follow-up was addressed | n/a. |
|  |  | (*e*) Describe any sensitivity analyses | n/a. |
| Results | | |  |
| Participants | 13* | (a) Report numbers of individuals at each stage of study—eg numbers potentially eligible, examined for eligibility, confirmed eligible, included in the study, completing follow-up, and analysed | Pag. 12. “Between January 2021 to March 2023, 64 consecutive patients were enlisted for lung transplantation at our Institution. Among them, 44 met the study’s inclusion criteria (see Fig 1). Among these, 10 (23%) patients had poor acoustic windows for RV echocardiographic evaluation (see Table S1, Online Supplement), resulting in 34 patients included in the analysis (see Table 1).” |
|  |  | (b) Give reasons for non-participation at each stage | Pag. 12. “Patients with poor acoustic windows showed a higher asymmetric pulmonary scintigraphy (p=0.002, OR 0.87 (0.76-0.99) compared to those with good acoustic windows. Challenging acoustic windows were primarily found in patients with thoracic and mediastinal anatomical alterations.” |
|  |  | (c) Consider use of a flow diagram | Figure 1. |
| Descriptive data | 14* | (a) Give characteristics of study participants (eg demographic, clinical, social) and information on exposures and potential confounders | Table 1, and table S1. |
|  |  | (b) Indicate number of participants with missing data for each variable of interest | Pag. 12 “No missing data was documented in the cohort of included patients.” And Table S1 |
|  |  | (c) Summarise follow-up time (eg, average and total amount) | Pag. 12. |
| Outcome data | 15* | Report numbers of outcome events or summary measures over time | n/a. |
| Main results | 16 | (*a*) Give unadjusted estimates and, if applicable, confounder-adjusted estimates and their precision (eg, 95% confidence interval). Make clear which confounders were adjusted for and why they were included | Pag. 12-13-14 |
|  |  | (*b*) Report category boundaries when continuous variables were categorized | n/a |
|  |  | (*c*) If relevant, consider translating estimates of relative risk into absolute risk for a meaningful time period | √ |
| Other analyses | 17 | Report other analyses done—eg analyses of subgroups and interactions, and sensitivity analyses | n/a |
| Discussion | | |  |
| Key results | 18 | Summarise key results with reference to study objectives | √ |
| Limitations | 19 | Discuss limitations of the study, taking into account sources of potential bias or imprecision. Discuss both direction and magnitude of any potential bias | Pag. 14 |
| Interpretation | 20 | Give a cautious overall interpretation of results considering objectives, limitations, multiplicity of analyses, results from similar studies, and other relevant evidence | Pag. 14-15-16 |
| Generalisability | 21 | Discuss the generalisability (external validity) of the study results | Pag. 17 |
| Other information | | |  |
| Funding | 22 | Give the source of funding and the role of the funders for the present study and, if applicable, for the original study on which the present article is based | Pag. 7 |

*Give information separately for exposed and unexposed groups.
