# Supplementary material for: Right ventricle free wall longitudinal strain screening of lung transplant candidates
Source: PLoS One. 2024 Dec 20;19(12):e0314235. doi: 10.1371/journal.pone.0314235 (PMC11661623; doi:10.1371/journal.pone.0314235)
Supplement: S1 File — (DOCX) [file pone.0314235.s002.docx]

**Title of article**

Right Ventricle Free Wall Longitudinal Strain Screening of Lung Transplant Candidates **Supplementary Material**

**Additional Methods**

**Lung Transplantation Management**

*LUTX Team*

Lung transplantation (LUTX) team at Fondazione IRCCS Ca' Granda - Ospedale Maggiore Policlinico is composed of two thoracic surgeons, one thoracic surgery fellows, a cardiac surgeon, two certified anesthesiologists, one thoracic anesthesia fellow, and one anesthesia fellow, a perfusionist, an operatory-room nurse and one surgical-nurse.

*LUTX Anesthetic preparation*

After routine monitor as per ASA guidelines, induction of general anesthesia with fentanyl (1-2 mcg/kg), midazolam (1-2 mg) and propofol (1-3 mg/kg), and muscle paralysis with rocuronium (0.6 mg/kg) is achieved. Patients are intubated with large size single-lumen tube and undergo aggressive bronchoscopic toilette. Then, the single-lumen tube is substituted, and lung isolation is achieved with appropriately sized left-sided double-lumen endotracheal tube under bronchoscopic guidance. General balanced anesthesia is maintained with fentanyl (1-2 mcg/kg/hr) and sevoflurane (end-tidal concentration 0.5%-1.0%), and muscle paralysis with sequential rocuronium boluses (0.15 mg/kg). Patients are monitored with invasive right radial artery cannulation, central venous catheterization of the right internal jugular vein, and oximetric pulmonary artery catheterization capable of cardiac output monitoring by means ofthermodilution technique. Trans-esophageal echocardiography is implemented pending the anesthesiologist preferences. A large bore (i.e., 7 Fr) catheter is introduced in the right antecubital vein and connected to a custom made rapid infusion system, capable of the infusion of up to 500 mL/min.Throughout the procedure, patients are mechanically ventilated in volume control mode, with FiO_2_ to maintain SpO_2_> 90%, and minute ventilation to limit hypercapnia and acidosis.

Antibiotic prophylaxis is provided following the indication of infectious disease specialists and pre-operative airways cultures. Immunosuppression comprises methylprednisolone 1000 mg and tacrolimus or basiliximab pending the patient renal function.

*LUTX Surgical procedure*

Patients with CF are treated with sequential bilateral LUTX, using bilateral anterolateral thoracotomy with a transverse sternotomy or two anterior thoracotomies, pending the anatomical characteristics of the patients. After lysis of pleural and mediastinal adhesions, the vascular structures and bronchi are isolated. Then the less perfused lung (as per pulmonary perfusion scintigraphy) is disconnected from the mechanical ventilation and allowed to deflate. The pulmonary artery is cross-clamped, and the first cross-clamping 10 minutes test is performed: the surgical procedure is halted, hemodynamics are strictly monitored,and blood gas analyses are obtained every 2 minutes.

The veins, artery, andbronchus are sequentially resected. The graft is positioned in the thoracic cavity, and the bronchial, arterial and venous anastomoses are created. The lung is thoroughly de-aired before vascular unclamping to avoid systemic air emboli; the first lung graft is slowly re-perfused and connected to another mechanical ventilator. The graft is initially ventilated in pressure control mode with FiO_2_ 21%, PEEP of 10 cmH_2_O, RR of 4 bpm and plateau pressure of 25 cmH_2_O. A recruitment maneuver is applied to obtain complete lung inflation. Progressively, ventilation and oxygenation of the graft are increasedto allow contralateral lung separation from ventilation. Particular attention is paid in limiting 1) FiO_2_ (i.e., < 50%); 2) driving pressures (i.e., < 15 cmH2O) and 3) de-recruitment (i.e., PEEP > 10 cmH_2_O) of the implanted graft. Then, contralateral native lung ventilation is interrupted, and the second pulmonary artery cross-clamping test is performed. Pneumonectomy of the second native lung and implantation of the second graft follows the procedure above. Finally, hemostasis is achieved, bilateral pleural drainage positioned, the thorax closed and the patient is transferred to the intensive care unit for follow-up.

*ECMO indication and pulmonary artery cross-clamping test.*

The pulmonary artery cross-clamping test is performed to simulate the hemodynamic conditions occurring during pneumonectomy in a controlled -and reversible- fashion, allowing the anesthesiologist to optimize hemodynamics and ventilation and eventually the cardiac surgeon to implement central veno-arterial ECMO in a semi-elective condition. During the first pulmonary artery cross-clamping test (while the native lung is ventilated and perfused), whether 1) pulmonary hypertension (i.e., systolic pulmonary artery pressure > 80 mmHg, 2) increase in PAPs > 50 mmHg associated with systemic hypotension (i.e., systolic arterial pressure < 60 mmHg) resistant to inotropic support; 3) major cardiac arrhythmias; 4) hypoxia (i.e., PaO_2_< 60 mmHg, despite increasing FiO_2_ up to 100% and optimizing PEEP); 5) respiratory acidosis (i.e., pH < 7.25 despite increasing minute ventilation) the test is interrupted and ECMO implemented.

During the second pulmonary-artery cross-clamping (while the graft is ventilated and perfused), our policy is more protective towards hyperoxia and ventilator-induced lung injury. Thus, during the second test FiO_2_ is not increased > 50% and driving pressure is not increased> 15 cmH_2_O. If one of the mentioned conditions occur, ECMO is implemented.

During all the surgical procedure, intractable hypoxemia, acidosis, and hemodynamic failure may occur at any given moment, but 1) single lung ventilation, 2) cross-clamping tests and 3) reperfusion of the grafts are the most critical. Whether during the procure 1) pulmonary hypertension (i.e., systolic pulmonary artery pressure > 80 mmHg, 2) increase in PAPs > 50 mmHg associated with systemic hypotension (i.e., systolic arterial pressure < 60 mmHg) resistant to inotropic support; 3) major cardiac arrhythmias; 4) hypoxia (i.e., PaO_2_< 60 mmHg, despite increasing FiO_2_ up to 100% to the native lung or 50% to the grafts and optimizing PEEP); 5) respiratory acidosis (i.e., pH < 7.25 despite increasing minute ventilation, while guaranteed driving pressure to the grafts < 15 cmH_2_O); the procedure is briefly halted, and ECMO implemented.

Our approach to intraoperative extracorporeal life support consists of central veno-arterial ECMO. After providing unfractionated heparin (i.e., 5000 UI) and eventual further boluses to achieve an aPTT>40 seconds, the ascending aorta and right atrium are cannulated. Blood is drained via a centrifugal pump directly to a polypropylene membrane lung where blood is oxygenated, decarboxylated, warmed and then directed to the central venous circulation. Initially, blood flow is set to achieve around 50% of the patient's cardiac output, gas flow to maintain normocapnia and fraction of oxygen in the sweep gas flow to maintain SpO_2_> 95%. The extracorporeal circuit setting is dynamically modified during the procedure, pending the different surgical and anesthetic requirements and mean arterial pressure is maintained > 60 mmHg by increases in extracorporeal blood flow, but complete blood drainage and emptying of the heart is avoided, and the opening of the aortic valve is always guaranteed.

At the end of the surgical procedure, prior to chest closure, patients undergoing VA-ECMO undergo a progressive de-escalation of extracorporeal support, consisting of: 1) reduction of blood flows down to 1 L/min; 2) reduction of extracorporeal FiO2 down to 21%. Hemodynamics are monitored and blood gas analysis are collected serially, to verify the patient do not suffer from: 1) hemodynamic failure/right ventricle failure; 2) hypoxemia; 3) acidosis and hypercapnia and/or need to ventilate the patients with tidal volume > 6 mL/kg or driving pressure > 14 cmH2O. Whether hemodynamic failure is observed, central VA-ECMO is converted to peripheral VA support (i.e., femoral cannulations). Whether hypoxemia and/or hypercapnia is observed, VV-ECMO (i.e., femoral-femoral cannulation) is instituted and prolonged in the post-operatory period.

No predefined standard management of blood components is applied, but patient-tailored transfusion management is carried out following blood gas analyses, point of care (POC) PT/aPTT tests, and thromboelastography, as per national guidelines.

*Postoperative Management*

We follow generally accepted guidelines for the postoperative management of LUTX recipients (see Di Nardo et al., Anesthesiology, 2022).

Immunosuppressive therapy comprises standard triple-drug therapy, including calcineurin inhibitors (i.e., tacrolimus), cell-cycle inhibitors (i.e., azathioprine), and corticosteroids (i.e., intraoperative bolus followed by maintenance).

Protective mechanical ventilation is guaranteed. We limit tidal volume to 6 mL/kg of donor-predicted body weight and plateau pressure < 30 cmH_2_O. We use the lowest inspired oxygen fraction capable of providing an arterial oxygen saturation > 90%. Positive end-expiratory pressure (PEEP) is set following a decremental PEEP trial, with the usual initial PEEP being >= 10 cmH2O. Higher levels of PEEP are utilized in case of plasmorrhea and/or respiratory failure. Possible step-up therapies employed in case of primary graft dysfunction are prolonging sedation, introducing neuromuscular blockade, and ECMO, as previously mentioned. We pursue early weaning from mechanical ventilation, and thus the standard non-complicated patient is usually rapidly weaned from mechanical ventilation and extubated in the first 24 hours after graft reperfusion employing elective noninvasive ventilation. A dedicated respiratory therapist is available 12 hours/24 hours, 7 days/7 days to tailor the management of noninvasive support.

Hemodynamic management is guided by lactate, urinary output, invasive cardiac output monitoring, pulmonary artery pressure, wedge pressure, and mixed venous saturation measurement through an elective pulmonary artery catheter. We do not employ a specific hemodynamic protocol but follow the following general rules of thumb: a restrictive fluid strategy to maintain euvolemia, high-threshold red blood cell transfusions (i.e., > 9 gr/L), and vasopressors are used to guarantee a mean arterial pressure in the 65-75 mmHg range and cardiac index (i.e., 2.2-2.5 L/min/m^2^).

Antimicrobial therapy is tailored to patients' and donors' characteristics. All patients receive prophylaxis for opportunistic infections: fungal (i.e., voriconazole), pneumocystis jirovecii (i.e., trimethoprim/sulfamethoxazole), and viral (i.e., ganciclovir). All patients receive surgical site infection prophylaxis for G- strains (i.e., cefepime for 72 hours) and G+ strains (i.e., vancomycin, one-shot). Cefepime is interrupted at 72 hours whether the donors' respiratory cultures are negative. Colonized patients continue their antibiotic treatment throughout the perioperative period. Strict infection surveillance is carried out during the postoperative period, by means of deep respiratory cultures collected at 24 hours, 5 days, and 15 days after LUTX and respiratory, anal, and perineal swabs for multi-drug resistant bacteria.

**Additional Results**

|  | | **Good RV Window** | **Poor RV Window** | **p** | **OR (95% CI)** |
| --- | --- | --- | --- | --- | --- |
| Gender (Male) | | 20 (59%) | 5 (50%) | 0.621 | 1.42 (0.34-5.88) |
| Age at enlistment (years) | | 48.0 [36.0-59.0] | 49.0 [40.5-59.8] | 0.689 | 0.98 (0.93-1.01) |
| Weight (kg) | | 62.5 [44.8-72.3] | 66.0 [52.8-77.5] | 0.469 | 0.98 (0.94-1.02) |
| Height (cm) | | 169 [158-175] | 164 [156-176] | 0.896 | 1.00 (0.93-1.07) |
| BMI (kg/m^2^) | | 22.5 [18.2-25.8] | 23.4 [19.3-28.2] | 0.282 | 0.91 (0.77-1.07) |
| BSA (m^2^) | | 1.7 [1.4-1.8] | 1.7 [1.5-1.9] | 0.624 | 0.48 (0.02-9.09) |
| Diagnosis | COPD | 9 (26%) | 0 (0%) | 0.129 | -- |
|  | Cystic Fibrosis/bronchiectasis | 10 (29%) | 3 (30%) |  |  |
|  | Hypersensitivity pneumonitis | 1 (3%) | 1 (10%) |  |  |
|  | Other | 2 (6%) | 1 (10%) |  |  |
|  | Primary Pulmonary Hypertension | 0 (0%) | 1 (10%) |  |  |
|  | Pulmonary Fibrosis | 12 (35%) | 4 (40%) |  |  |
| Pulmonary Fibrosis + other | | 14 (41%) | 5 (50%) | 0.621 | 0.70 (0.16-2.88) |
| Lung Allocation Score | | 38.2 [34.9-42.6] | 36.3 [34.9-41.6] | 0.662 | 1.02 (0.91-1.14) |
| O_2_ need at rest | | 1.0 [0.8-2.0] | 1.5 [1.0-2.3] | 0.454 | 0.84 (0.55-1.29) |
|  | FVC (% predicted) | 49.0 [38.8-68.0] | 49.5 [35.8-58.5] | 0.501 | 1.01 (0.97-1.04) |
|  | FEV_1_ (% predicted) | 28.5 [18.8-40.3] | 35.0 [26.3-57.5] | 0.415 | 0.98 (0.95-1.01) |
|  | DLCO (% predicted) | 23.0 [13.8-36.0] | 18.5 [4.8-23.8] | 0.105 | 1.04 (0.98-1.09) |
| 6 Minutes Walking Test (mt) | | 383 [252-457] | 386 [345-441] | 0.673 | 0.99 (0.99-1.00) |
| Arterial Blood Gas Analyses at rest | FiO_2_ at rest | 24.0 [21.0-28.0] | 28.0 [23.3-28.5] | 0.988 | 0.99 (0.89-1.11) |
|  | pH | 7.4 [7.4-7.5] | 7.4 [7.4-7.5] | 0.202 | 7.2 (0.31-166.2) |
|  | pO_2_ (mmHg) | 76.5 [69.0-88.5] | 83.0 [70.0-103.3] | 0.085 | 0.97 (0.94-1.00) |
|  | pCO_2_ (mmHg) | 44.0 [37.8-51.3] | 43.0 [38.0-52.8] | 0.859 | 0.99 (0.93-1.06) |
|  | HbO_2_ (%) | 95.2 [94.0-98.0] | 95.0 [94.0-99.0] | 0.666 | 0.93 (0.67-1.28) |
| Cardiac Catheterization | CO (L/min) | 4.8 [4.3-6.2] | 5.6 [3.5-6.0] | 0.580 | 1.17 (0.65-2.12) |
|  | CI (L/min/m^2^) | 3.0 [2.6-3.3] | 3.5 [1.8-3.9] | 0.918 | 1.05 (0.40-2.70) |
|  | HR (bpm) | 73.5 [65.5-87.8] | 83.0 [75.0-90.0] | 0.352 | 0.97 (0.91-1.03) |
|  | PAPm (mmHg) | 21.0 [18.0-24.0] | 22.0 [11.5-36.0] | 0.899 | 0.99 (0.92-1.07) |
|  | Pw (mmHg) | 9.0 [6.0-10.3] | 9.0 [4.0-11.0] | 0.724 | 1.04 (0.83-1.30) |
| Pulmonary hypertension | | 7 (23%) | 4 (44%) | 0.210 | 0.36 (0.07-1.73) |
| Pulmonary Scintigraphy (% left lung) | | 47.0 [36.4-51.0] | 57.0 [49.6-61.0] | **0.002** | **0.87 (0.76-0.99)** |
| RV Ejection Fraction (%) | | 49 [45-62] | 53 [43-65] | 0.336 | 0.95 (0.88-1.04) |
| RV Ejection Fraction < 40% | | 5 (15%) | 1 (10%) | 0.752 | 1.42 (0.14-14.0) |

**Table S1. Patients' characteristics (good RV window vs. poor RV window).**

Data are presented as absolute frequency (% of the included patients) or as median and interquartile range. OR, odds ratio; CI, confidence interval. BMI, body mass index; BSA, body surface area; COPD, chronic obstructive pulmonary disease; FEV_1_, 1st second forced expiratory volume; FVC, forced vital capacity; DLCO, diffusing capacity of the lungs for carbon monoxide; pO_2_, Oxygen Partial Pressure; pCO_2_, carbon dioxide partial pressure; HbO_2_, hemoglobin saturation; CO, cardiac output; CI, cardiac index; HR, heart rate; PAPm, mean pulmonary artery pressure; Pw, wedge pressure; RV, right ventricle. For continuous variables the OR per unit in change regressor is showed.

**Figure S1. Linear correlations between right ventricle global longitudinal strain and right ventricle free wall longitudinal strain.**


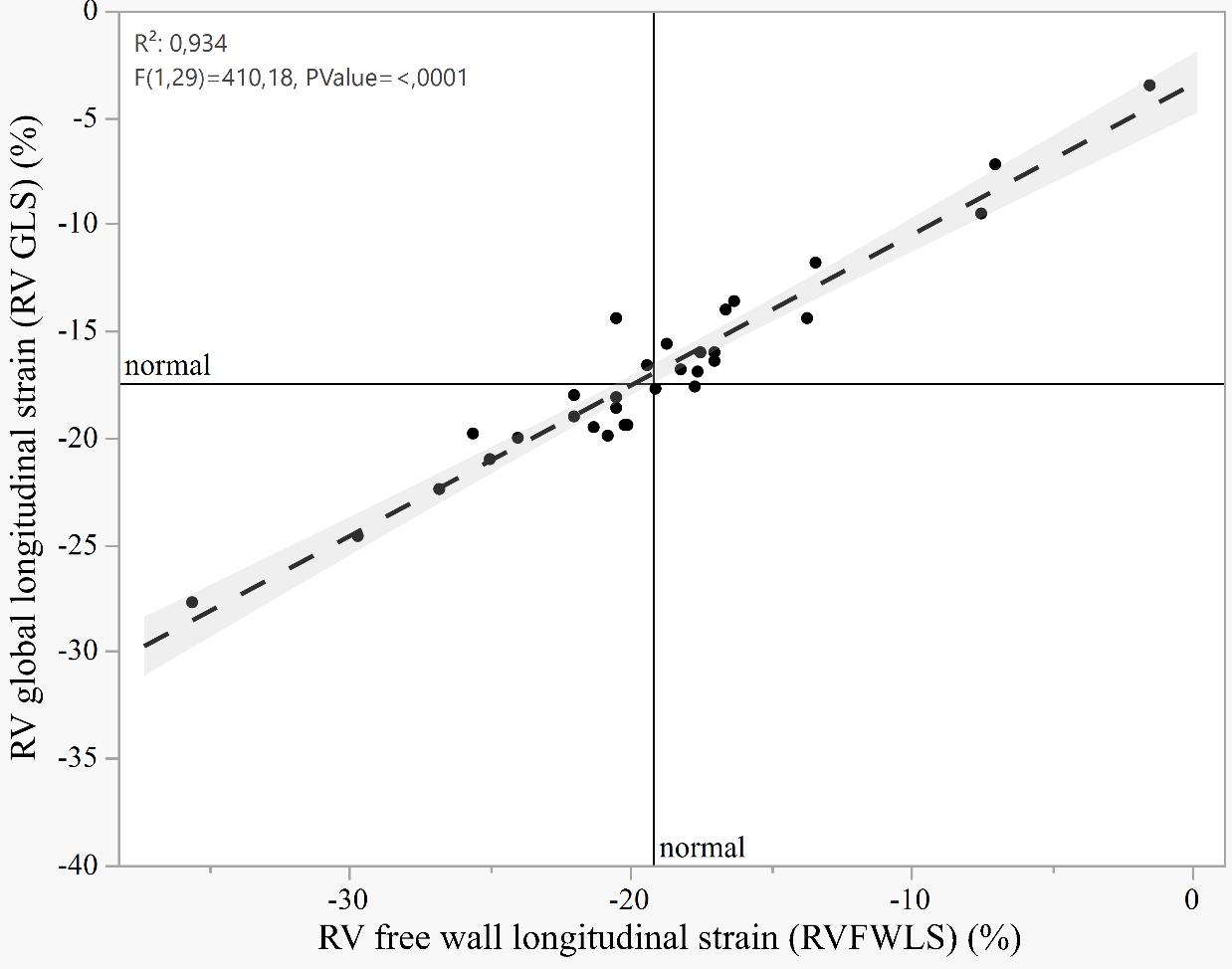


RV, right ventricle; RVFWLS, right ventricle free wall longitudinal strain; RV GLS, right ventricle global longitudinal strain.

**Figure S2. Linear correlations between right ventricle systolic strain and right ventricle ejection fraction (measured by multi-gated radionuclide ventriculography).**


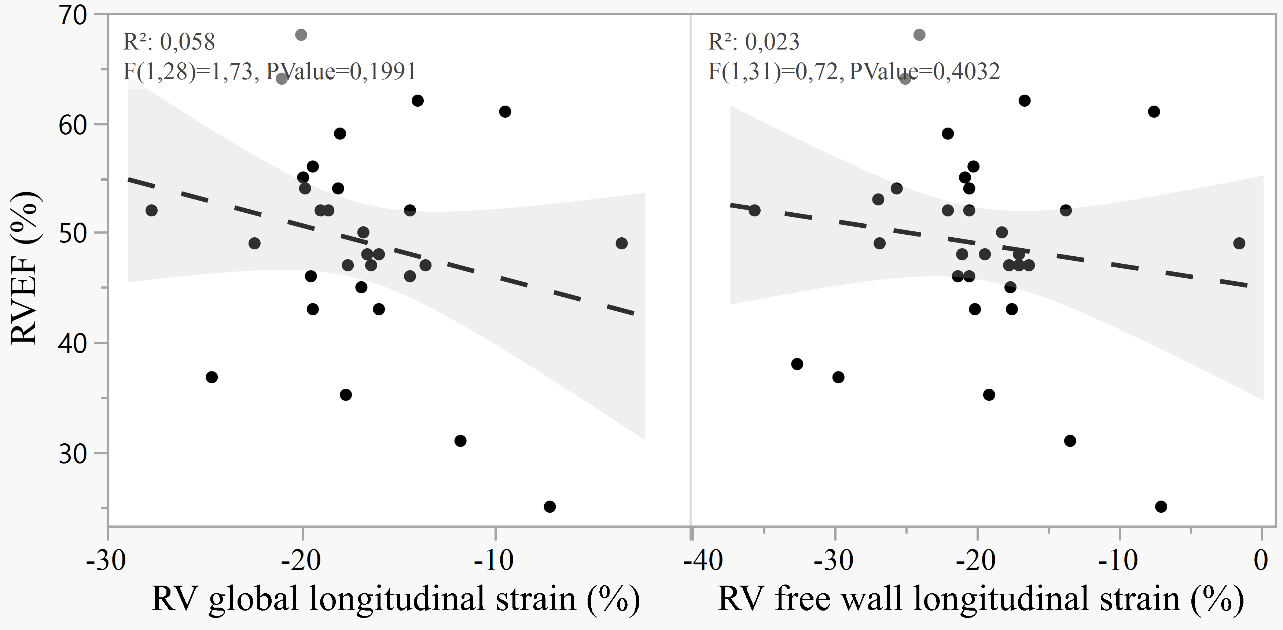


RVEF, right ventricle ejection fraction; RV, right ventricle.

**Figure S3. Linear correlations between RV free wall longitudinal strain (RVFWLS) and invasive right heart catheterization measurements.**

**
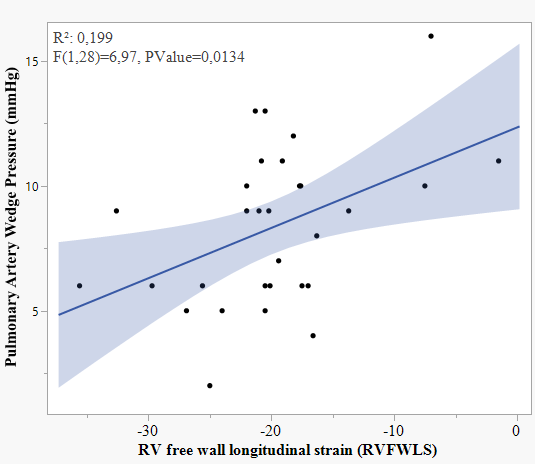

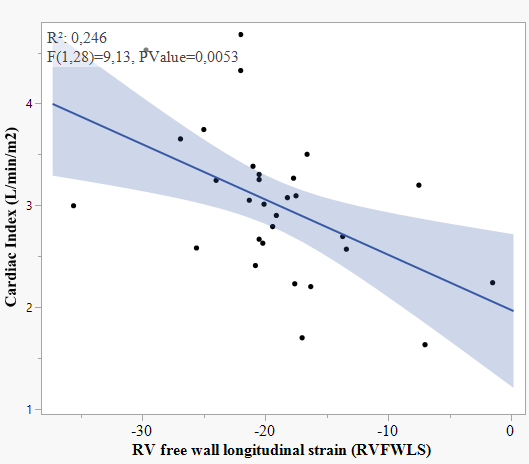
**

**
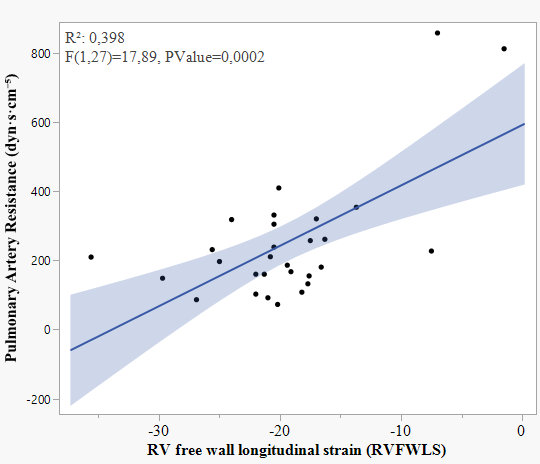

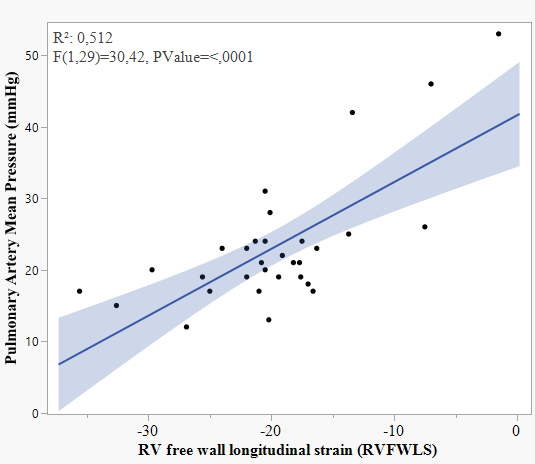

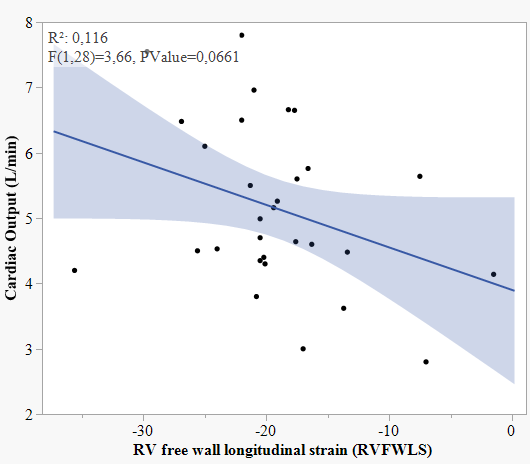
**

**
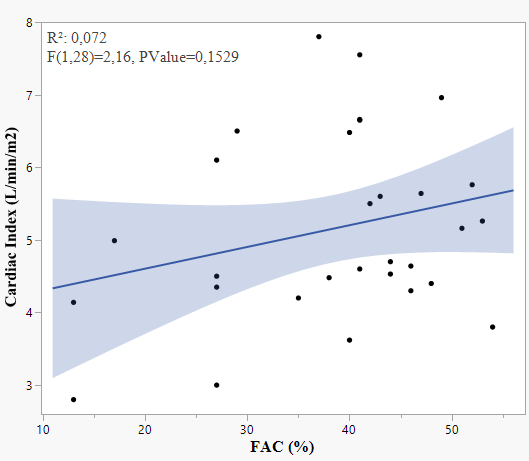

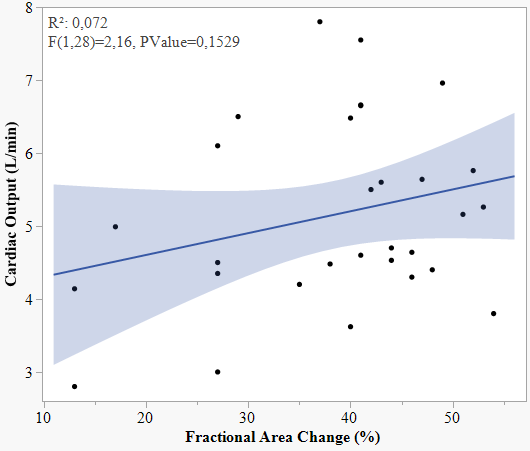
Figure S4. Linear correlations between Fractional Area Change (FAC) and invasive right heart catheterization measurements.**

**
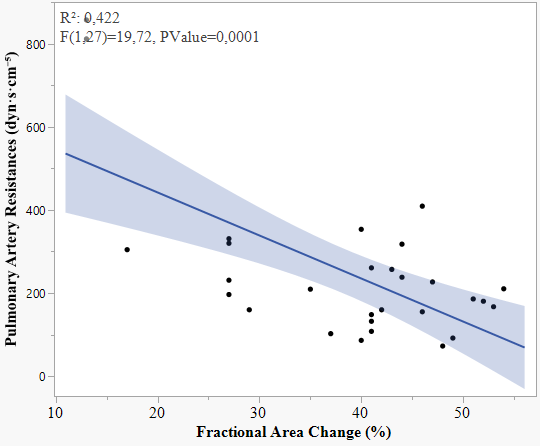

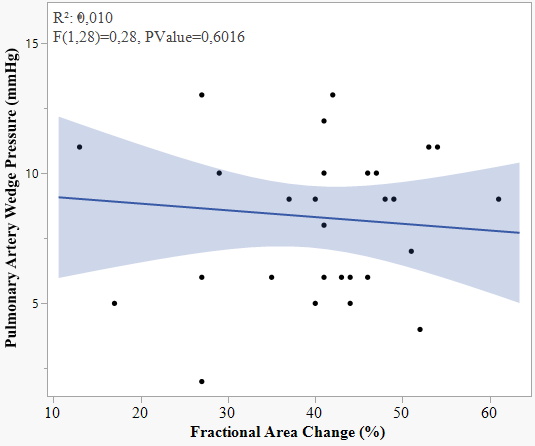

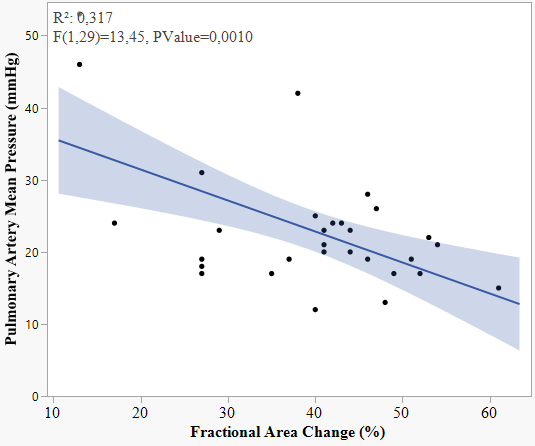
**

**
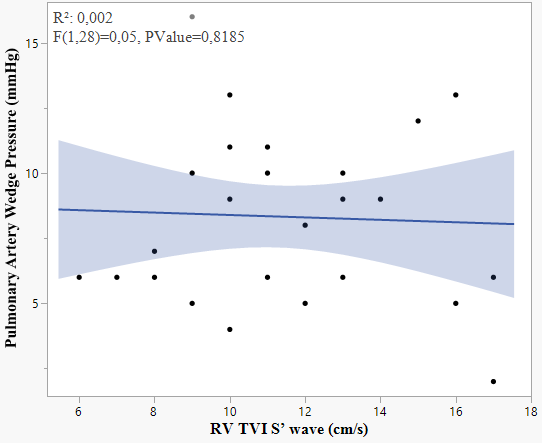

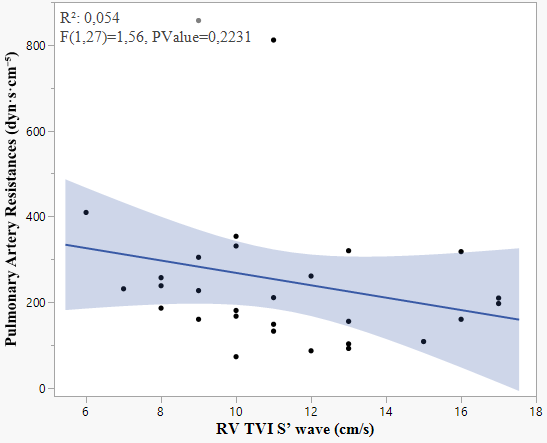

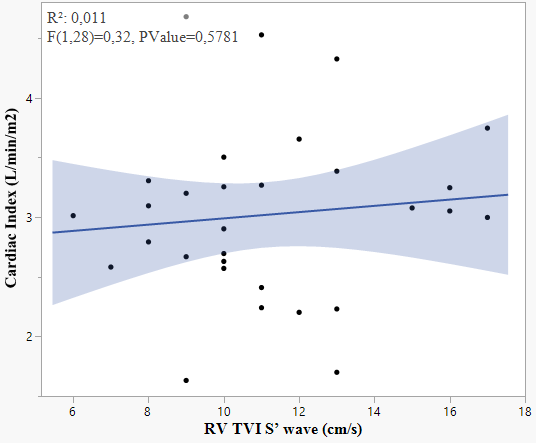

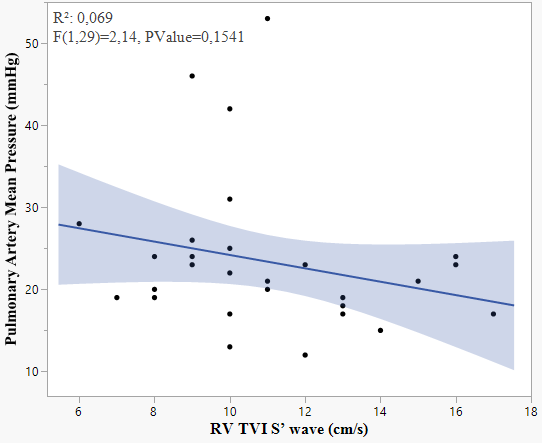

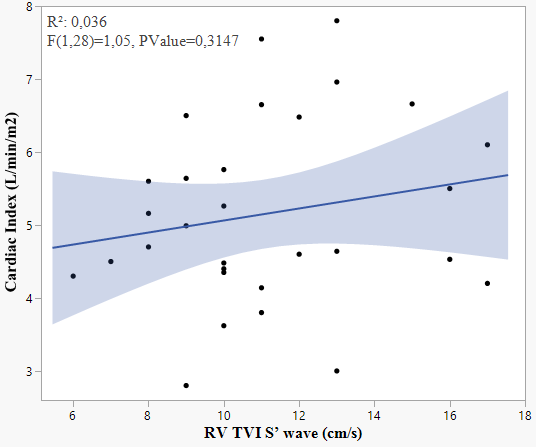
Figure S5. Linear correlations between S', tissue Doppler positive peak systolic wave velocity and invasive right heart catheterization measurements.**

**
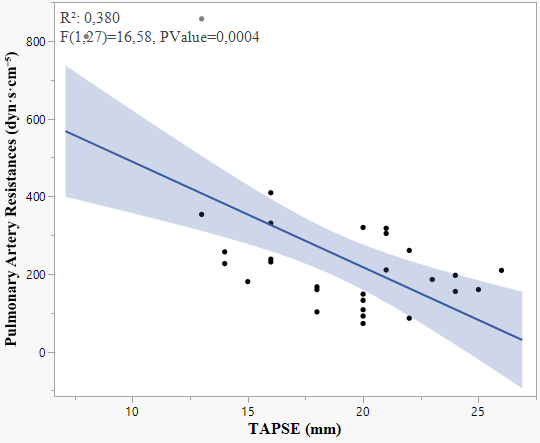

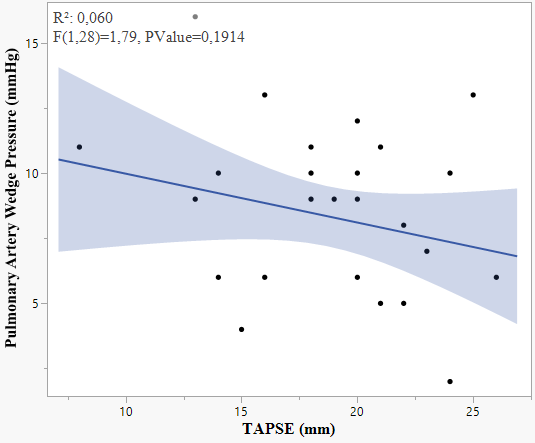

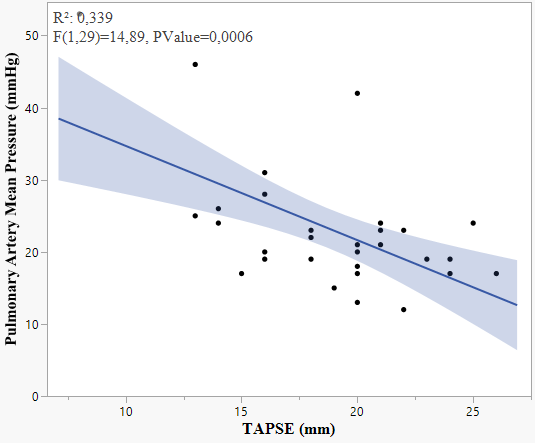

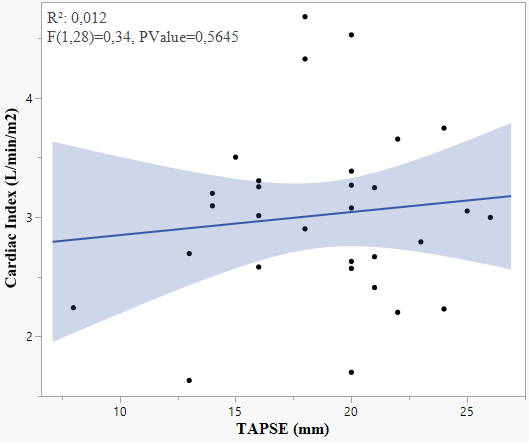

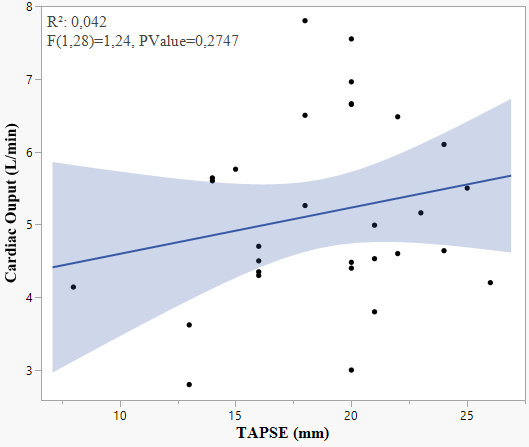
Figure S6. Linear correlations between tricuspid annular plane systolic excursion (TAPSE) and invasive right heart catheterization measurements.**
