# Supplementary material for: Right ventricle free wall longitudinal strain screening of lung transplant candidates
Source: PLoS One. 2024 Dec 20;19(12):e0314235. doi: 10.1371/journal.pone.0314235 (PMC11661623; doi:10.1371/journal.pone.0314235)
Supplement: S2 File — (DOC) [file pone.0314235.s003.doc]

# **“RIGHT VENTRICLE DYSFUNCTION IN PATIENTS UNDERGOING LUNG TRANSPLANT”**

**Study Code: LUXT STRAIN**

Version 2.0 – November 9, 2021

**Promoter:** Fondazione IRCCS Ca’ Granda Ospedale Maggiore Policlinico via F. Sforza 28, 20122 Milano

**Coordinator:** UOC Anestesia, Rianimazione e Emergenza Urgenza, Fondazione IRCCS Ca’ Granda Ospedale Maggiore Policlinico

**Principal Investigator:** Vittorio Scaravilli, M.D.

**Signature:**

**Study Design:** observational single-center, prospective, non-pharmacological, no profit

**1. LIST OF ABBREVIATIONS**

**2. TIMELINE OF THE STUDY**

**3. BACKGROUND**

**4. HYPOTHESIS**

**5. SUBJECT POPULATION**

5.1 Study type

5.2 Inclusion criteria

5.3 Exclusion criteria

**6. PROTOCOL**

**7. ENDPOINTS**

**8. BENEFITS**

**9. STATISTICAL METHODS**

**10. STUDY MANAGEMENT**

10.1 Data collection and handling

1. 10.2 Ethical considerations
2. 10.2.1 Ethical considerations and regulatory standards

10.2.2 Institutional review board / independent ethics committee

10.2.3 Patient information

10.3 Finance

10.4 Insurance

10.5 Confidentiality

10.5.1 Source document requirement – Confidentiality

10.5.2 Publication

**11. BIBLOGRAPHY**

**1. List of abbreviations**

CBP: cardiopulmonary bypass

CF: Cystic Fibrosis

CRF: Case Report Form

ECLS: extracorporeal life support

ECMO: extracorporeal membrane oxygenation

GCP: Good Clinical Practice

IEC: Independent Ethics Committee

LUTX: lung transplant

PGD: primary graft dysfunction

PI: Principal Investigator

RV: Right Ventricle

RVLS: right ventricle longitudinal strain

**2. Timeline of the study**

Starting date: October 2019

Closing of Enrollment: December 2022

Closing date: March 2023

**3. Background**

Patients enlisted for bilateral lung transplantation (LUTX) have subclinical right ventricle (RV) dysfunction^1^, which is usually clinically silent until LUTX. During LUTX, several reasons (i.e., sequential pulmonary arteries cross-clamp, hypoxia, hypercapnia) lead to de-compensation of RV function, cardiac failure and shock^2^. In this clinical scenario, extracorporeal life support (ECLS) with cardiopulmonary bypass (CBP) or extracorporeal membrane oxygenation (ECMO) is emergently implemented.

ECLS is associated with prolonged mechanical ventilation, primary graft dysfunction (PGD), bleeding, and graft rejection^3^. This may be due to: 1) the activation of pro-inflammatory cascade due to blood-circuit contact; 2) the increased need for allogenic blood components, which per se has been associated to an increased risk of PGD^4^.

Avoiding intraoperative ECLS may thus have significant positive clinical outcomes. In the general cohort of patients undergoing LUTX, pulmonary hypertension, and right ventricular dysfunction have been identified as risk factors for intraoperative ECLS^5^.

At enlistment for LUTX, patients undergo a comprehensive evaluation of right cardiac function comprising: transthoracic echocardiography, pulmonary artery catheterization, and calculation of RV ejection fraction (RVEF) by multiple gated radionuclide ventriculography. Echocardiography is non-invasive, can be performed repeatedly and at the bedside. To the contrary, pulmonary artery catheterization and ventriculography are invasive, may have severe complications and thus are performed only once at enlistment. In a recent retrospective analysis of 82 CF patients undergoing LUTX at our institution, we observed that patients with depressed RVEF (measured by ventriculography) had increased risk for intraoperative ECLS use.

The free-wall RV longitudinal strain (RVLS) is a novel echocardiographic method for quantification of myocardial deformation^6^ with high diagnostic accuracy to predict depressed RV ejection fraction. RVLS may be used for non-invasive, repeated and bedside assessment of RV function before, during (i.e., using transesophageal echography) and after LUTX. We envision the employment of RVLS to document subclinical RV dysfunction before LUTX, and screen patients at high risk of intraoperative RV failure which would need ECLS to carry out LUTX.

**4. Hypothesis**

Patients enlisted for LUTX have subclinical right heart failure which is associated with intraoperative cardiac failure and need for ECLS. The use of ECLS is associated with worse short- and long-term outcomes. In patients undergoing LUTX, clinicians need an early, non-invasive, repeatable diagnosis of RV dysfunction, to allow prevention and timely perioperative treatment. With this study, we hypothesize RVLS to be a reliable predictive index for intraoperative cardiac failure and subsequent ECLS use.

**5. Subject population**

**5.1 Study type**

Prospective observational single-center non-pharmacological study.

**5.2 Inclusion criteria:**

- Enlistment for bilateral LUTX
- Age > 18 years
- Signed informed consent

**5.3 Exclusion criteria**:

- Age < 18 years old
- Urgency enlistment
- Already undergone LUTX
- Extracorporeal membrane oxygenation (ECMO) bridging to LUTX
- Poor acoustic windows which limit the adequate acquisition of the echocardiographic

pictures

- Congenital heart disease
- Previous cardiac surgery

**6. Protocol**

Demographic and clinical data

The clinical course of the patients will not be altered by the participation to the study, since:

1) the included patients will not undergo any further invasive test due to the study; 2) with the technology available at our Institution, the RVLS can be calculated exclusively offline and will be performed by a cardiologist blinded to treatment of the patients *ex post*, after drop-out of the patients from the study (i.e., 3 months follow-up, see later).

The following clinical characteristics and data at the time of enlisting for lung transplant will be collected: demographics, weight, height, lung allocation score (LAS), arterial blood gas analysis, spirometry; oxygen at rest; six-minute walking test (6MWT); comprehensive trans-thoracic echocardiography; invasive cardiac catheterization; pulmonary scintigraphy; multiple gated radionuclide ventriculography.

The following intra-operative data will be collected: waiting list time; use of ECLS for LUTX, type (i.e., CBP or ECMO) and cannulation (i.e., peripheral or central) of ECLS; the reason for ECLS utilization (i.e., hemodynamic or respiratory failure); timing of ECLS cannulation (i.e., first or second graft); length of surgery; length of intraoperative ECLS support; use of blood components; use of inotropic drugs and relative dosages; occurrence of unexpected intraoperative complications (i.e., cardiac arrest, occurrence of arrhythmias, allergic reactions).

The following outcomes will be collected: length of ECLS support; need for ECLS at end of surgery; length of mechanical ventilation; intensive care unit (ICU) length of stay (LOS); ICU use of inotropic drugs and relative dosages; hospital LOS; PGD grade at 72 hours from reperfusion; survival at 30 days; need for surgical revision; survival at 3-months.

Measurements

Echocardiographic examinations will be performed using a GE Vivid IQ machine (GE Healthcare, Milwaukee, WI). Images will be acquired during breath holds with stable electrocardiographic recordings and will be digitally stored for subsequent offline analysis using EchoPAC Clinical Workstation Software (GE Healthcare, Milwaukee, WI). Echocardiography will be performed as per standard clinical practice (i.e., at enlistment for LUTX, pre-operative, intra-operative, at ICU admission, at 3-months follow-up), and RVLS will be calculated ex-post using conventional the two-dimensional echocardiographic apical 4-chamber view images^7,8^.

Figure 1. Speckle tracking strain imaging.

In post-processing analysis, the region of interest will be obtained by tracing the RV endocardial borders at the level of the septum and the free wall in a still frame at end-systole. An automated software program will calculate the displacements of the speckle pattern within the region of interest throughout the cardiac cycle^9^. Longitudinal strain curves will be obtained for 6 RV segments (the basal, mid, and apical segments of the RV free wall and septum), and the global RV regional strain curve. A certified sonographist will perform all echocardiographic examinations and measurements. A certified cardiologist will review all post-processing measurement.

**7. Endpoints**

This study has the aim of:

- assessing whether RVLS is a predictive index of the need for intraoperative ECLS need;
- validating RVLS as compared to multiple gated radionuclide ventriculography.

**8. Benefits**

Whether our hypothesis will be confirmed, the care of patients undergoing LUTX could be improved since we will develop a new index for identification of patients at high risk for intraoperative deterioration and need for ECLS.

**9. Statistical methods**

All the patients enlisted for LUTX will be considered for inclusion.

As previously mentioned, we recently carried out a retrospective analysis describing the incidence of right ventricle failure during LUTX for CF patients. We included 82 adult CF patients and observed that CF patients with RV ejection fraction (measured by ventriculography at enlistment) < 40% had a high risk of ECLS (i.e., 66%), while CF patients with RV ejection fraction >40% had a low risk of ECLS (i.e., 33%). Moreover, a ratio of 1:2 was observed between high-risk and low-risk patients. A recent paper from Sciatti et al.^10^ studying RVLS in CF patients has been recently published. A significant reduction in RV strain has been demonstrated in adult CF patients, as compared to the healthy population (-23.4 ± -7.6 vs. -30.4 ± -3.3 non-CF patients). Combining these and the data obtained from our preliminary retrospective analysis, we were able to calculate sample size and power of the analyses (using the G power 3.1.9.4 program), as follows.

For a chi-square test, with an α = 0.05 and β = 0.8, a sample size of 25 patients would be sufficient to discriminate a population at high risk (i.e., 66%) and low risk (i.e., 33%) for the need of intraoperative extracorporeal membrane oxygenation (see Figure 2).


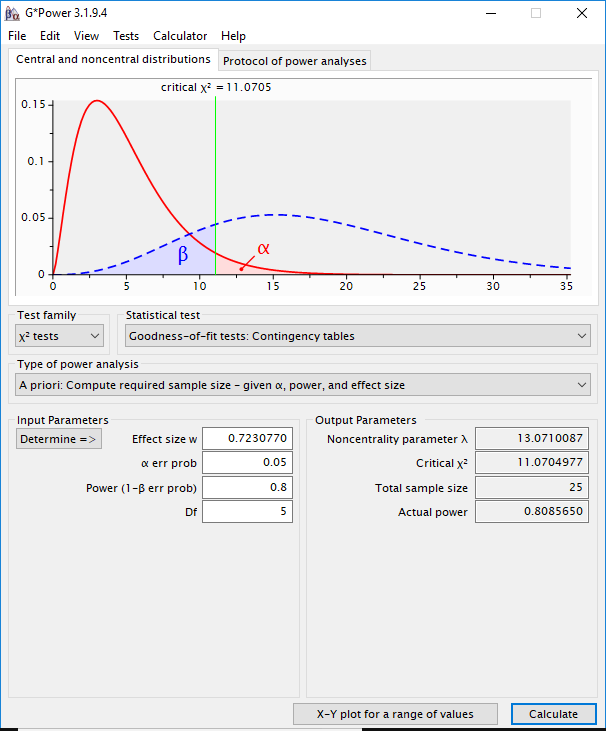

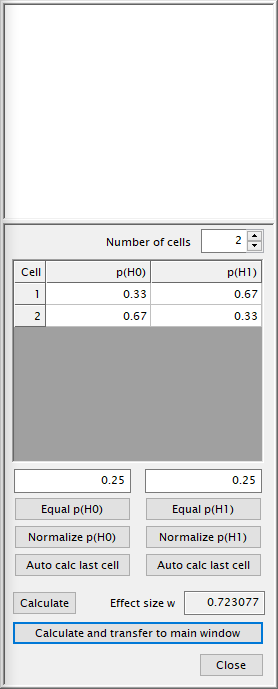


Figure 2. Sample size estimate for the study (G*power 3.1.9.4).

Assuming that: 1) high-risk patient and low-risk patients to have RVLS of -23.4 ± -7.6 and -30.4 ± -3.3, respectively; 2) low to high-risk patients ratio to be maintained as in our observational study (2:1); 3) with α = 0.05 and 4) with 30 included patients, the β power for a t-test would be 0.91.


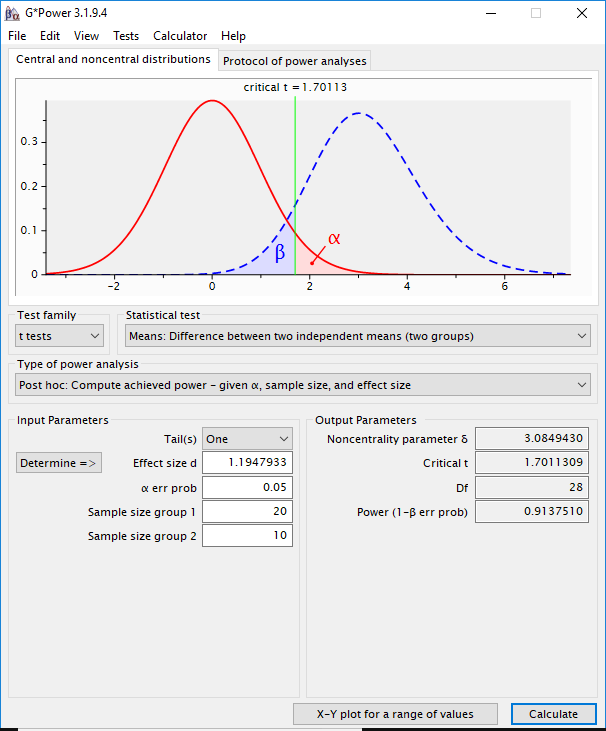

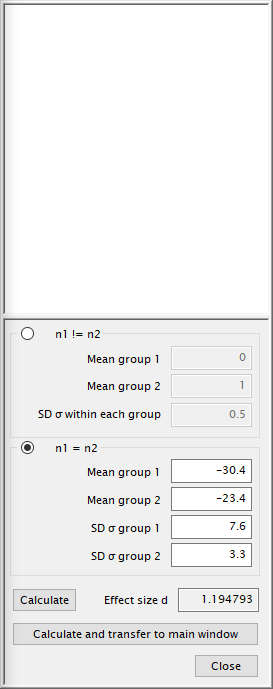


Figure 2. β power estimate for Aim 1 study (G*power 3.1.9.4).

Thus, to observe a clinically meaningful difference in RVLS between high-risk and low-risk cohorts, 30 patients would be sufficient. At our Institution, roughly 30 patients undergo LUTX each year. Thus, we consider appropriate a length of study of 2 years, in view of a possible exclusion rate of 25%.

Data will be reported as the median and interquartile range for continuous variables. Categorical variables will be expressed as the number of patients. RV longitudinal strain will be described with standard descriptive statistics. The goodness of fit between enlistment RVLS echocardiographic measurements and RV ejection fraction obtained by multi-gated radionuclide ventriculography will be assessed with the R^2^ coefficient, linear regression analyses, and sensitivity/sensibility analysis. Association between RVLS measurements and use of intraoperative ECLS will be assessed by logistic regression: odds ratios (OR) and associated 95% likelihood ratio based confidence intervals will be calculated, and comparison between ECLS and non-ECLS patients will performed with the chi-square test or the Fisher’s test, as appropriate. The cutoff value for RVLS that will be sensitive and specific in predicting the need for intraoperative ECLS will be determined by means of receiver operating characteristic curve analysis. All statistical tests will be 2-tailed, and statistical significance will be accepted at P < 0.05.

**10. Study management**

**10.1 Data collection and handling**

Demographic data, clinical profiles, laboratory data and therapeutic regimens of the patients will be extracted from the clinical documents and recorded in a protected database. Case Report Forms (CRF) will be designed by the PI. A unique code will be assigned to each participant in order to de-identify the data. It is the Investigator’s responsibility to ensure the accuracy of all data entered and recorded in the CRFs.

Data concerning demographics, pre-existing medical conditions/medications, clinical parameters will be entered directly in the CRF for research-only investigations. Echocardiographic data will be entered on to the CRF after analysis of the imagery has been conducted. Demographic, clinical, and echocardiographic data will be entered into a protected database. The database will be password protected and stored on a password-protected PC in a research office in the critical care department.

Data analysis will be the responsibility of the PI.

1. **10.2 Ethical considerations**
2. **10.2.1. Ethical considerations and regulatory standards**
3. This study will be conducted in accordance with Good Clinical Practice (GCP) rules; in accordance to the ethical principles that have their origin in the Declaration of Helsinki and with the respect to the European clinical practice; in compliance with all international guidelines and national law regulation in Italy.
4. **10.2.2. Institutional review board / independent ethics committee**
5. The protocol and the informed consent document must be submitted to the Independent Ethics Committee (IEC) for review and will receive IEC approval/favorable opinion before initiation of the study. During the study, any amendments to the protocol must also be approved by IEC. A progress report is sent to the IEC at least annually and a summary of the study’s outcome is send at the end of the study.
6. **10.2.3. Patient information**

Patients enrolled will be required to consent to the anonymous use of personal data for the study. Information form and the module for the acquisition of informed consent for the handling of sensitive data will be given to the patient.

The investigator will fulfill the current regulations for research and documentation of informed consent, the standards of Good Clinical Practice, and the ethical principles derived from the Declaration of Helsinki. The approval by the Ethics Committee will be required whether an update of the informed consent form will be needed during the study,

According to the recommendations of the Declaration of Helsinki and local regulations, each patient will be adequately informed about the aims, methods, expected benefits, potential risks and problems related to the study. Moreover, patients will be informed of their right to refuse consent to the use of their sensitive data or to withdraw it at any time, without having any effect on their medical care.

The patient will have all the time necessary for the evaluation of the information received before providing their informed consent to the use of sensitive data. The investigator will have to obtain spontaneous informed consent in writing by the patient before using them in any way for the study. The written consent to the handling of sensitive data must be subscribed by the date and signature of the patient and by the investigator's or his representative's ones.

The investigator has to give the patient a signed copy of informed consent; the original form will be retained with the other documents of the study protocol; the module for the acquisition of informed consent to the treatment of sensitive data will be attached to the clinic folder. The collaborator will be appointed to review the original forms of all patients’ informed consents.

**10.3. Finance**

No additional costs for the Institution are planned as all the exams are part of the standard clinical practice. To now, all the aforementioned echocardiographic technology is available at our center.

## 10.4. Insurance

The internal institutional insurance policy will cover for any undesirable effects due to the participation in the study.

## 10.5. Confidentiality

**10.5.1 Source document requirement - Confidentiality**

According to the ICH guidelines for the Good Clinical Practice, the monitoring team must check the CRF entries against source documents. The personnel bound by professional secret must maintain the confidentiality of all personal identity or personal medical information (according to the confidentiality and personal data protection rules). The confidentiality of records that could identify subjects should be protected, only initials of the name and the first name will be registered with a inclusion coded number for the study (no name nor address nor identifying data).

**10.5.2. Publication**

Communications, reports, and publication of the results of the study will be under the responsibility of the principal investigator of the study. A summary of the results of the study will be written and provided on request of the participating patients.

## 11. Bibliography

1. Ionescu AA, Ionescu A, Payne N, Obieta-fresnedo I, Fraser AG, Shale DJ. Subclinical Right Ventricular Dysfunction in Cystic Fibrosis A Study Using Tissue Doppler Echocardiography. 2001;(5).

2. Triantafillou AN, Pasque MK, Huddleston CB, et al. Predictors, frequency, and indications for cardiopulmonary bypass during lung transplantation in adults. *Ann Thorac Surg*. 1994;57(5):1248-1251. doi:10.1016/0003-4975(94)91367-6.

3. Hoechter DJ, Shen YM, Kammerer T, et al. Extracorporeal Circulation during Lung Transplantation Procedures: A Meta-Analysis. *ASAIO J*. 2017;63(5):551-561. doi:10.1097/MAT.0000000000000549.

4. Diamond JM, Lee JC, Kawut SM, et al. Clinical risk factors for primary graft dysfunction after lung transplantation. *Am J Respir Crit Care Med*. 2013;187(5):527-534. doi:10.1164/rccm.201210-1865OC.

5. Shah PR, Boisen ML, Winger DG, et al. Extracorporeal Support During Bilateral Sequential Lung Transplantation in Patients With Pulmonary Hypertension: Risk Factors and Outcomes. *J Cardiothorac Vasc Anesth*. 2017;31(2):418-425. doi:10.1053/j.jvca.2016.08.021.

6. Meris A, Faletra F, Conca C, et al. Timing and magnitude of regional right ventricular function: a speckle tracking-derived strain study of normal subjects and patients with right ventricular dysfunction. *J Am Soc Echocardiogr*. 2010;23(8):823-831. doi:10.1016/j.echo.2010.05.009.

7. Toyoda T, Baba H, Akasaka T, et al. Assessment of regional myocardial strain by a novel automated tracking system from digital image files. *J Am Soc Echocardiogr*. 2004;17(12):1234-1238. doi:10.1016/j.echo.2004.07.010.

8. Badano LP, Kolias TJ, Muraru D, et al. Standardization of left atrial, right ventricular, and right atrial deformation imaging using two-dimensional speckle tracking echocardiography: a consensus document of the EACVI/ASE/Industry Task Force to standardize deformation imaging. *Eur Heart J Cardiovasc Imaging*. 2018;19(6):591-600. doi:10.1093/ehjci/jey042.

9. Teske AJ, De Boeck BW, Melman PG, Sieswerda GT, Doevendans PA, Cramer MJ. Echocardiographic quantification of myocardial function using tissue deformation imaging, a guide to image acquisition and analysis using tissue Doppler and speckle tracking. *Cardiovasc Ultrasound*. 2007;5(1):27. doi:10.1186/1476-7120-5-27.

10. Sciatti E, Vizzardi E, Bonadei I, et al. Focus on echocardiographic right ventricular strain analysis in cystic fibrosis adults without cardiovascular risk factors: a case–control study. *Intern Emerg Med*. 2019. doi:10.1007/s11739-019-02104-5.
