# Supplementary material for: Right ventricle free wall longitudinal strain screening of lung transplant candidates
Source: PLoS One. 2024 Dec 20;19(12):e0314235. doi: 10.1371/journal.pone.0314235 (PMC11661623; doi:10.1371/journal.pone.0314235)
Supplement: Attachment — Submitted filename: Response to reviewers.docx [file pone.0314235.s006.docx]

PONE-D-23-43919

Right Ventricle Free Wall Longitudinal Strain Screening of Lung Transplant Candidates

PLOS ONE

**Response to Statistical Reviewer**

Dear Editorial Office,

We thank you all for the support, and the revisions provided. Hereby you will find a point by point response to the Statistical Reviewer.

As the statistical reviewer I will focus on methods and reporting. this is the first time i see the paper.

1) Question #1. the power calculations are appropriate and replicable. My concern is why noit use multiple imputation if you have missing data, so you can increase power a little bit - plus it's a better analytical aproach? how many cases in total were dropped becaise of a complate case anlaysis been conducted?

1) Response #1. Thank you for the comment. We appreciate your suggestion to use multiple imputation. However, given the exploratory nature of our study and our limited sample size, we believe complete case analysis was more appropriate. Multiple imputation can introduce additional uncertainty, particularly in small samples with complex data structures. Our analysis focused on maintaining transparency about missing data and providing a clear, conservative interpretation of results. While multiple imputation might marginally increase statistical power, in an exploratory study, we prioritized methodological clarity and avoiding potential over-interpretation of imputed data. Moreover, as clearly documented in the text, the only missing data was the actual measure of RVFWLS (which was the focus of the study), and was not measurable in 10 over 44 patients, as follows:

“Among them, 44 met the study's inclusion criteria (see Fig 1). Among these, 10 (23%) patients had poor acoustic windows for RV echocardiographic evaluation (see Table S1, Online Supplement), resulting in 34 patients included in the analysis (see Table 1). No missing data was documented in the cohort of included patients.”

2) Question #2. how were the confdence intervals for sensitivity specificity etc computed? bootstrapping? please state.

2) Answer #2. Thank you for the comment. The confidence intervals were calculated using the Wilson approach. We added a reference for the interested reader.

3) Question #3. discuss the low power of the logistic regression as a limitation as well as of most analyses except the primary research question - the confidence intervals obtained are very wide.

3) Answer #3. We acknowledge the limitations of statistical power in our analysis. The wide confidence intervals reflect the exploratory nature and small sample size of our study. These limitations are inherent to our research design and are directly related to the pilot/preliminary nature of our investigation. We added this limitation to the Limitations section of the paper, as follows:

“*We acknowledge the limitations of statistical power in our analysis, leading to ample confidence intervals reflecting the exploratory nature and small sample size of our study. Further, more populated studies are necessary to confirm our findings. Again*, …”

4) Question #4. language correction are needed e.g. robust logistic regression -> implying statistical significance. "was tested with the R^2 logistic regression" does not make sense some words are missing. Also note that logistic regression returns a pseudo-R^2 which is debatable how interpretable it it.

4) Answer #4. We carried out a thorough language revision to avoid any misleading implication. Our analysis did not involve logistic regression pseudo-R² calculations. R² values were exclusively derived from continuous variable associations, as stated in the phrase “The correlation between continuous variables was tested with the R2 linear regression.”
